# Step-Up Management in Acute Pancreatitis: A Tertiary Care Center's Experience From Southern India

**DOI:** 10.7759/cureus.58971

**Published:** 2024-04-25

**Authors:** Joel Kumar Earjala, Thiruvarul Muthukumarasamy, Senthil Kumaran Govindaraj Raman, Kalyanasundarabharathi V. C., Mathews Micheal, Vivek G Nath, Arun Raja A., U. Aravindan

**Affiliations:** 1 Surgical Gastroenterology and GI Oncology, Thanjavur Medical College, Thanjavur, IND; 2 Surgical Gastroenterology, All India Institute of Medical Sciences, Bhubaneswar, IND

**Keywords:** necrosectomy, necrotizing pancreatitis, severe acute pancreatitis, step-up approach, acute pancreatitis

## Abstract

Background

The clinical spectrum of acute pancreatitis (AP) ranges from mild disease to severe form associated with multiorgan failure, prolonged hospital stay, high morbidity, and mortality. Acute necrotizing pancreatitis (ANP) is a severe form of AP. This study evaluates AP's outcomes after applying principles of the *step-up* approach in a tertiary healthcare center in south India.

Methodology

This prospective observational study was carried out from January 2021 to December 2022. The study population includes patients admitted to our department with AP.

Results

Ninety patients were included in the study, most of them were middle-aged males with ethanol ingestion as the common etiology. Thirty-seven (41.1%) patients had mild AP, 25 (27.7%) had moderately severe AP, and 28 (31.1%) had severe AP. Organ failure at admission was noted in 36 (40%) patients. Twenty-three (25.5%) patients developed ANP. Infected necrosis was noted in 3 (3.33%) patients. Eighteen (20%) patients needed image-guided percutaneous drainage. Seven (38.8%) needed necrosectomy following percutaneous drainage. Mortality was observed in 8 (8.8%) patients. Specifically, mortality was noted in 6 (6.6%) patients who presented later in their disease course.

Conclusions

Percutaneous catheter drainage is a safe and effective therapy to tide over the initial phase of AP. It also serves as a bridging therapy till the patient is clinically fit for a necrosectomy. Severe AP cases presenting late in their course are associated with significant mortality even after *step-up *management. Standardized protocols for referral and management are essential to obtain a good clinical outcome.

## Introduction

Acute pancreatitis (AP) is an inflammatory process of the pancreas predisposed to various causative factors. The clinical spectrum of AP ranges from mild disease resolving with supportive therapy to severe form associated with systemic inflammatory response syndrome (SIRS) and subsequent organ dysfunction requiring intensive care unit (ICU) management and prolonged hospital stay, morbidity, and mortality. The mortality rate in severe cases can be as high as 30% [1].

Various severity scores are available at present to assess the severity of AP. Grading severity of AP is prognostic and directly correlates with the need for ICU care, nutritional support, and interventions. Acute necrotizing pancreatitis (ANP) is a severe form of AP with necrosis of pancreatic parenchyma and peripancreatic tissues.

In the PANTER trial, a minimally invasive *step-up* approach led to a lower rate of major complications and death compared with primary open necrosectomy [2].

There is a paucity of multicentric Indian data regarding pancreatitis. This study is undertaken to evaluate the various aspects of presentation, evaluation, and management outcomes of AP after applying principles of the *step-up* approach in a tertiary healthcare center in south India.

## Materials and methods

Study design

This is a prospective observational study conducted in the Department of Surgical Gastroenterology at Thanjavur Medical College and Hospital, Thanjavur, Tamilnadu, India. The study was conducted from January 2021 to December 2022. The study population included patients admitted to the Department of Surgical Gastroenterology with the diagnosis of AP during the study period. All the patients were prospectively followed up through the course of the hospital stay, and various parameters were assessed as per the study protocol. The study was approved by the Institutional Ethics Committee, and prior informed and written consent was obtained from all the study participants.

Inclusion criteria 

Patients aged >15 years and <70 years with AP presenting to our department were included in this study.

Exclusion criteria 

Patients aged less than 15 years with a diagnosis of AP were excluded.

Management protocol 

All patients presenting with clinical features and history suggestive of AP undergo initial serum amylase and lipase level assessments, in addition to baseline blood investigations, including complete blood counts, renal function tests, serum electrolytes, arterial blood gas analysis, C-reactive protein levels, and ultrasound of the abdomen and pelvis performed upon admission. After a clinical diagnosis of AP (pain in upper abdomen radiation to back with elevated serum lipase three times the institutional normal value), medical management is initiated with 150-250 mL/hour of Ringer lactate in the first 24-48 hours. For patients presenting with hypovolemia or shock, 1500 mL initial intravenous (IV) fluid bolus is given over 30 minutes for one or two cycles. In patients with persistent hypotension, vasopressor support is started along with additional fluid boluses as needed after a thorough assessment of circulatory status, aiming for a target mean arterial pressure of >65 mmHg. Frequent reassessment is done to achieve desired mean arterial pressure >65 mmHg, urine output >0.5 mL/kg, and capillary refilling time <3 seconds. Patients are monitored for signs of fluid overload, and the fluid infusion rate is titrated to achieve a neutral fluid balance. Supportive therapy for organ failure is administered as required, with vasopressors, oxygen supplementation, ventilatory support, and dialysis.

Contrast-enhanced pancreatic protocol computerized tomography (CT) scan is performed after 72 hours in all cases. The severity of AP is graded according to the Revised Atlanta classification [3]. All cases of moderately severe and severe pancreatitis are kept under ICU monitoring. Patients are reassessed after 72 hours.

Enteric nutrition is initiated as early as possible whenever the patient's clinical condition permits. Nasogastric or nasojejunal feeding is provided if necessary. Total parenteral nutrition (TPN) is started for patients in whom enteric nutrition is contraindicated (hemodynamic instability and paralytic ileus). IV fluids are tapered in patients who tolerate adequate oral intake for >24 hours.

Patients not responding to initial medical management are stepped up with ultrasound or CT-guided percutaneous catheter drainage (PCD) in cases with drainable acute pancreatic fluid collections (APFCs) or acute necrotic collection (ANC). Prophylactic broad-spectrum antibiotics are started. Drained collections are subjected to culture and sensitivity, and antibiotic therapy is tailored accordingly. The patient is then monitored for 72 hours for response and improvement in biochemical, clinical, and vital parameters. If there is no clinical improvement of organ failure or systemic inflammatory response syndrome (SIRS) features, repeat imaging using either contrast-enhanced CT or ultrasound of the abdomen is performed. Residual collections/inadequate drainage via existing PCD are managed by an additional PCD placement or repositioning of existing PCD. After further ICU management, patients with no clinical improvement, persistent organ failure, or failure to thrive are stepped up and proceeded for minimally invasive or open necrosectomy, preferably after four weeks of clinical course. Patients with features suggestive of infected necrosis on imaging are drained with PCD immediately and followed up according to the aforementioned protocol. Patients improving clinically are stepped down and discharged with PCD in situ if necessary and followed up.

Data collection and analysis

Patients were enrolled in the study after admission and diagnosis of AP and were prospectively followed up during their hospital stay. Prospective patient data were compiled after the study duration and analyzed. Qualitative variables were represented with percentages and tables. Fisher's exact test was used to examine the significance of moderate and severe AP in local and systemic complications and mortality based on the time of admission.

## Results

Patient demographics

A total of 90 patients were admitted with acute pancreatitis and treated in our institution during the study period. Patients were in the age group of 23-61 years (mean ± SD 39.8 ± 10.7 years). Of these, 83 patients were males and 7 were females. Body mass index ranged between 17.3 and 34.3 (mean ± SD 26.3 ± 4.5). The time of presentation ranged from 1 day of onset of symptoms to 14 days (mean ± SD 7 ± 4.5). The most common etiology in our study was ethanol ingestion (80, 88.8%), followed by biliary etiology (6, 7.5%), hyperlipidemia (1, 1.25%), autoimmune causes (1, 1.25%), and idiopathic AP (2, 2.5%). Most of the cases of AP are initially managed at secondary healthcare centers by specialty departments and referred to us in due course depending on the severity of AP for expert management. Thirty-two (35.5%) patients were referred from other centers (Table 1). 

**Table 1:** Patient characteristics. BMI, body mass index; SD, standard deviation

Characteristics	*n *= 90 (%)
Age	
Range (years)	23-61
Mean ± SD (years)	39.8 ± 10.7
Males	83 (92.2)
Females	7 (7.7)
BMI	
Range	17.3-34.3
Mean ± SD	26.3 ± 4.5
Time of presentation (days)	1-14
Mean ± SD	7 ± 4.5
Etiology	
Alcoholic	80 (88.8)
Biliary	6 (6.6)
Hyperlipidemia	1 (1.1)
Autoimmune	1 (1.1)
Idiopathic	2 (2.2)
Revised Atlanta grading	
Mild	37 (41.1)
Moderate	25 (27.7)
Severe	28 (31.1)
Referred from another center	32 (35.5)

Revised Atlanta classification grades

Based on the Revised Atlanta grading system [3], 37 (41.1%) patients were categorized as having mild acute pancreatitis(MAP), 25 (27.7%) as having moderately severe acute pancreatitis (MoSAP), and 28 (31.1%) as having severe acute pancreatitis (SAP). SIRS features were observed in 63 (70%) patients at the time of admission (24, 64.8%, cases of MAP; 16, 64%, cases of MoSAP; and 23, 82.14%, cases of SAP). Persistent SIRS after 48 hours since initial management was seen in 34 (37.7%) cases (5, 13.5%, cases of MAP; 9, 36%, cases of MoSAP; and 20, 71.4%, cases of SAP). Organ failure at admission was noted in 36 (40%) patients. The organ system most commonly involved was the respiratory system (26, 28.8%) followed by the renal system (14, 15.5%) and cardiovascular system (9, 10%). Transient organ failure(<48 hours) was noted in eight cases of MoSAP. New-onset organ failure developed in 7 (31.1%) patients with SAP during the 7- to 14-day period. Significant association was seen with acute fluid collection, necrotizing pancreatitis, transient organ failure, and persistent organ failure with the severity of AP. No significant association was seen between infected necrosis and SIRS at or after 48 hours with the severity of AP (Table 2).

**Table 2:** Local and systemic complications in severity groups. SIRS, systemic inflammatory response syndrome; MAP, mild acute pancreatitis; MoSAP, moderately severe acute pancreatitis; SAP, severe acute pancreatitis

	MAP (*n *= 37)	MoSAP (*n *= 25)	SAP (*n *= 28)	*P*-value
Acute fluid collection	0	15	12	<0.001
Necrotizing pancreatitis	0	7	16	<0.001
Infected necrosis	0	2	1	0.45
SIRS at admission	24	16	23	0.48
SIRS at or after 48 hours	5	9	20	<0.001
Transient organ failure (48 hours)	-	8	0	<0.001
Organ failure				
Respiratory	-	5	21	<0.001
Renal	-	3	11	<0.001
Cardiovascular	-	0	9	<0.001

Pancreatic necrosis and infection

A total of 23 (25.5%) patients developed ANP. APFCs were observed in 27 (30%) patients. ANP is more commonly identified in patients with SAP (APFC, 9, 32.14%; ANP, 7, 25%) than in patients with MoSAP (APFC, 5, 20%; ANP, 2, 8%). Extra pancreatic infections were seen in 14 (15.5%) patients. The most common site is the lungs (9, 64.2%), followed by the urinary tract (7, 50%). Twenty-six (28.8%) patients needed prophylactic antibiotic therapy (4, 10.8%, patients of MAP; 7, 28%, patients of MoSAP; and 15, 53.5%, patients of SAP group). Infected necrosis was noted in 3 (3.33%) patients (2, 8%, patients in MoSAP and 1, 3.5%, patient in SAP group).

Management

The average amount of intravenous fluid therapy administered in the first 48-72 hours was 4 ± 2.5 L. The need for increased amounts of IV fluids was noted as the severity increased.

ICU admission and monitoring were done in all patients with MoSAP and SAP (53, 58.8%). Although all cases of MoSAP were admitted for ICU care, the duration of stay varied between MoSAP and SAP. Twelve (13.3%) patients developed fluid overload features during therapy. Nutritional support by TPN was needed in 20 (22.2%) patients of which 16 (57.1%) were SAP patients and 4 (16%) were MoSAP patients. Eight (8.8%) patients needed assisted enteric nutrition via nasojejunal tube (3, 12%, patients with MoSAP and 5, 17.8%, patients with SAP). 

A total of 60 (66.6%) patients were managed conservatively by medical management (37 patients with MAP, 18 patients with MoSAP, and 5 patients with SAP). A total of 18 (20%) patients needed intervention in the form of drainage or necrosectomy. Eighteen (20%) patients needed image-guided percutaneous catheter drainage (PCD) of APFC or infected necrotic collections. Seven (28%) patients with MoSAP and 11 (44%) with SAP were managed with PCD. Of the 11 patients with SAP who underwent PCD, 7 (7.7%) patients needed necrosectomy in their due course. Of the 53 patients with MoSAP and SAP, 2 (3.7%) patients underwent laparoscopic necrosectomy and 5 (9.4%) patients underwent open necrosectomy. Six of 11 (54.5%) patients in the SAP group with persistent renal insufficiency after 72 hours required hemodialysis. Twelve of 21 (57.1%) patients of SAP with respiratory failure required mechanical ventilator support (Table 3).

**Table 3:** Management outcomes. MAP, mild acute pancreatitis; MoSAP, moderately severe acute pancreatitis; SAP, severe acute pancreatitis; PCD, percutaneous catheter drainage; ICU, intensive care unit

	MAP (*n *= 37), *n* (%)	MoSAP (*n *= 25), *n* (%)	SAP (*n *= 28), *n* (%)
Conservative	37 (41.1)	18 (20)	10 (11.1)
Prophylactic antibiotics	4 (4.4)	7 (7.7)	15 (16.6)
Percutaneous drainage	0	7 (7.7)	11 (12.2)
Necrosectomy			
Minimally invasive	0	0	2 (2.2)
Open	0	0	5 (5.5)
Additional procedures with necrosectomy			
Diversion stoma	0	0	2 (2.2)
Feeding jejunostomy	0	0	5 (5.5)
Total parenteral Nutrition	0	4 (4.4)	16 (17.7)
Prophylactic antibiotics	4	7 (7.7)	15 (16.6)
Duration of ICU stay (days)	-	7-20	20-35
Total hospital stay (days)	4-7	10-28	21-45
PCD-related morbidity			
Pancreatic fistula	0	0	2 (2.2)
Enterocutaneous fistula	0	0	0
Bleeding	0	1 (1.1)	2 (2.2)
Mortality	0	1 (1.1)	7 (7.7)

Outcomes

The duration of hospital stay was four to seven days for MAP cases, 10-28 days for MoSAP cases, and 21-45 days for SAP cases. The duration of ICU stay was seven to 20 days for MoSAP cases and 20-35 days for SAP cases.

The most common morbidity after PCD placement was pancreatic fistula, which was observed in two of 18 (11.1%) patients who resolved spontaneously on observation. Three (16.6%) patients had bleeding from PCD and were managed conservatively. There were no PCD-related enterocutaneous fistulas in the study group.

The overall mortality rate was 8.8%, with 8 out of 90 patients. Severity-specific mortality was 1 of 25 patients (4%) in MoSAP and 7 of 28 patients (25%) in the SAP group. Mortality in all seven patients with SAP was due to overwhelming multi-organ failure. Five of 7 (71.4%) patients who had mortality in the SAP group expired in the early phase of treatment <5 days after admission.

**Figure 1:**
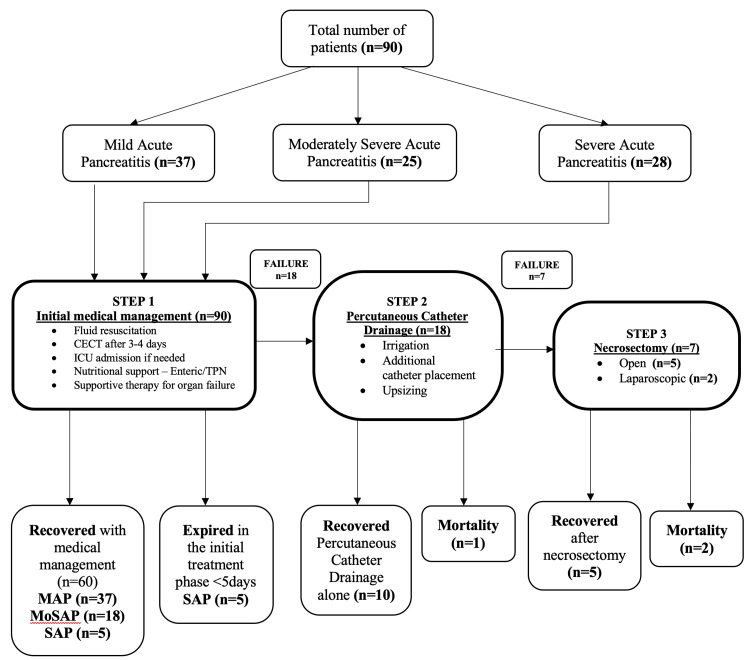
Study outline. Image credit: Earjala Joel Kumar. MAP, mild acute pancreatitis; MoSAP, moderately severe acute pancreatitis; SAP, severe acute pancreatitis; CECT, contrast-enhanced computerized tomography; ICU, intensive care unit; TPN, total parenteral nutrition

Of the total 53 (58.8%) cases of MoSAP and SAP managed during the study period, 32 (35.5%) patients were referred from other centers after seven days of onset of AP (range 10-15 days). Of the 17 (18.8%) cases of SAP admitted after seven days of onset, 8 (8.8%) patients needed intervention. Mortality was observed in 6 (6.6%) patients who presented later in the disease course. There was no statistically significant association noted between time of presentation and mortality in patients with MoSAP and SAP (Table 4; Figure 1).

**Table 4:** Outcomes based on severity of acute pancreatitis and time of presentation. MoSAP, moderately severe acute pancreatitis; SAP, severe acute pancreatitis

Severity of pancreatitis	Presentation	Total	Needed intervention	Mortality	*P*-value
MoSAP (*n *= 25)	<7 days	10	2	0	0.41
	>7 days	15	5	1	
SAP (*n *= 28)	<7 days	11	3	1	0.19
	>7 days	17	8	6	

## Discussion

In 2019, India had the highest incidence of AP in the world, with 618,862 cases and an age-standardized incidence of 40-60 per 100,000 population. In the same year, India was among the top 3 countries in terms of mortality associated with AP [4]. Early diagnosis and severity stratification are key for the effective management of AP. That being said, the mortality rates in SAP are still the same, emphasizing the need for strict implementation of validated diagnostic, therapeutic, and referral protocols for prompt management and better outcomes.

In our study, males were the predominant sex accounting for 92.2% of total patients. Ethanol-related AP was the predominant etiology in our study (88.8%), similar to the findings of Mukherjee et al. and Negi et al., from northern and eastern India, respectively [5,6]. Most of our patients were in the mean age of 37.4 ± 12.3 years in whom alcohol consumption was more prevalent. Similar findings were noted in two case series from southern India by Ramu et al. and Vengadakrishnan and Koushik [7,8]. Worldwide demographic data published by Matta et al. showed similar findings in the Indian population, while biliary pancreatitis was the most common etiology in the rest of the world [9].

After grading of severity based on the Revised Atlanta grading system, most of the cases in our series are MAP (37, 41.1%) [3]. Cases referred from secondary healthcare centers numbered 32 (35.5%), all of which involved patients with MoSAP and SAP. All the cases are managed by the institutional treatment protocol based on the American College of Gastroenterology (ACG) guidelines on AP and the step-up approach for AP as emphasized in the PANTER trial [2,10]. Initial fluid resuscitation in our patients was consistent with the ACG guidelines, 150-250 mL/hour of ringer lactate solution in the first 24-48 hours [10]. Of the total patients, 13.3% developed features of fluid overload in a consecutive period, which was less when compared with the WATERFALL trial, which showed 20.5% of fluid overload in the aggressive resuscitation arm [11]. Although it cannot be generalized, in our experience, the concept of initial aggressive fluid resuscitation still holds good for the initial phase of AP. Consequent fluid overload and its sequelae can be managed through prompt identification of signs of fluid overload, fluid titration to achieve a neutral fluid balance, and the provision of adequate supportive therapy for existing organ failure. Fluid therapy in patients with AP should be a delicately balanced act by judiciously applying the principles of aggressive and goal-directed fluid therapy.

The actual utility of the step-up approach comes into play in cases of MoSAP and SAP rather than MAP. Hence, our analysis of the results of outcomes will be confined to the 53 (58.8%) cases belonging to the MoSAP and SAP groups. Of the 53 cases, 23 (43.3%) were managed conservatively and 25 (27.7%) needed intervention. Of the 25 cases of MoSAP and 28 cases of SAP, 7 (28%) and 11 (64.2%) patients required intervention, respectively. The need for intervention is proportional to the severity of AP. Similar results were shown by Babu et al. with intervention rates of 71.7% in SAP patients [12]. 

Seventeen (32.07%) patients in our series needing intervention were managed by PCD alone. Our intervention rates are comparable with the PANTER trial (32.07% compared with 35%) and higher than that of Horvath et al. (32.07% compared to 23%) [2,13]. Most of these cases in our series showed a reversal of organ failure after PCD alone (26.3%). The most common PCD-related morbidity was pancreatic fistula, occurring in 11.7% (2/17) of patients, a rate lower than that reported in the PANTER trial (28%), as well as by Babu et al. (17.8%) and Horvath et al. (29%) [2,12,13].

Seven (12.06%) patients required necrosectomy after initial PCD. Persistent sepsis and failure to thrive are the most common indications for necrosectomy. Necrosectomy rates are lower in our series compared with a study by Babu et al. (38.5%) and the PANTER trial (60%) [2,12]. In our experience, necrosectomy is needed in cases where the necrosum is extensive and PCD of peripancreatic collections alone may not deliver the expected clinical improvement. 

In our series, the overall mortality rate is 8.8% (*n* = 8). When considering only the MoSAP and SAP groups, the mortality rate is 15.09% (*n* = 8).In comparison, the mortality rate in our series was lower than that reported in the PANTER trial (19%) and by Babu et al. (24.2%), but higher than that reported by Horvath et al. (2.5%) [2,12,13]. When considering patients with SAP, our mortality rate was 25%, which was higher compared to the multicentric experiences reported by De Rai et al. (20.7%) and Rasch et al. (13.6%) in the management of SAP [14,15].

In total, 60.3% (32/53) cases presented after seven days of onset of AP in MoSAP and SAP groups. All eight cases of mortality in our study group, presented to us late (>7 days) after undergoing their initial management at other centers. In our series, there was no statistically significant association between time of referral and mortality in patients with MoSAP and SAP. In our experience, patients presenting late in the disease course with established persistent organ failure and decompensation have a poorer outcome than their counterparts.

The assessment of the severity of AP at the time of presentation and prompt referral to a higher center with expertise and facilities for the management of AP is crucial and should be effectively implemented to improve the outcome in these patients. Hence, in our opinion treating physicians at the level of primary and secondary health care centers need to be aware of the wide spectrum of AP and recognize cases with SAP in the early stages, and there needs to be a low threshold to refer a patient with SAP and organ failure to centers of expertise. A standardized institutional protocol for the evaluation and management of AP is very essential to implement a step-up approach effectively and undertake the necessary clinical judgments.

Though prospective data and application of AP management protocol based on established guidelines are the strengths of our study, our limitations include a smaller sample size, nonrandomized data, and being a single institutional experience (Table 5).

**Table 5:** Comparison of our results with similar studies. PCD, percutaneous catheter drainage

	Present study	PANTER trial [2]	Horvath et al. [13]	Babu et al. [12]	De Rai et al. [14]	Rasch et al. [15]
Patients needing intervention with PCD	32.07%	35%	23%	-	-	-
Post-PCD pancreatic fistula	11.7%	28%	29%	17.8%	-	-
Patients needing necrosectomy	12.06%	60%	-	38.5%	-	-
Mortality	25%	19%	2.5%	24.2%	20.7%	13.6%

## Conclusions

Males with ethanol ingestion form the majority of cases of AP in our region. Most of the cases (23, 43.3%) of MoSAP and SAP can be managed conservatively without any interventions. PCD is a safe and effective therapy to tide over the initial phase of AP. It also serves as a bridging therapy until the patient is clinically fit for a necrosectomy. Cases of SAP must be identified in the very early phase and referred to centers of expertise for effective management and better outcomes. SAP cases presenting late are associated with significant mortality even after applying the principles of the step-up approach in their management. Hence, easy-to-use and validated severity scoring systems should be implemented at the peripheral centers to identify such patients for early referral. Standardized institutional protocols are essential for obtaining a good clinical outcome.
